# Hypertension and Exposure to Noise Near Airports: the HYENA Study

**DOI:** 10.1289/ehp.10775

**Published:** 2007-12-11

**Authors:** Lars Jarup, Wolfgang Babisch, Danny Houthuijs, Göran Pershagen, Klea Katsouyanni, Ennio Cadum, Marie-Louise Dudley, Pauline Savigny, Ingeburg Seiffert, Wim Swart, Oscar Breugelmans, Gösta Bluhm, Jenny Selander, Alexandros Haralabidis, Konstantina Dimakopoulou, Panayota Sourtzi, Manolis Velonakis, Federica Vigna-Taglianti

**Affiliations:** 1 Department of Epidemiology and Public Health, Imperial College London, St Mary’s Campus, Norfolk Place, London, United Kingdom; 2 Department of Environment and Health at the Federal Environmental Agency (UBA), Berlin, Germany; 3 National Institute of Public Health and Environmental Protection (RIVM), Bilthoven, the Netherlands; 4 Institute of Environmental Medicine (IMM), Karolinska Institutet, Stockholm, Sweden; 5 Department of Hygiene and Epidemiology, National and Kapodistrian University of Athens, Athens, Greece; 6 Environmental Epidemiologic Unit, Regional Agency for Environmental Protection (ARPA), Piedmont Region, Grugliasco, Italy; 7 Laboratory of Prevention, Nurses School, National and Kapodistrian University of Athens, Athens, Greece

**Keywords:** aircraft, blood pressure, hypertension, noise, road traffic

## Abstract

**Background:**

An increasing number of people are exposed to aircraft and road traffic noise. Hypertension is an important risk factor for cardiovascular disease, and even a small contribution in risk from environmental factors may have a major impact on public health.

**Objectives:**

The HYENA (Hypertension and Exposure to Noise near Airports) study aimed to assess the relations between noise from aircraft or road traffic near airports and the risk of hypertension.

**Methods:**

We measured blood pressure and collected data on health, socioeconomic, and lifestyle factors, including diet and physical activity, via questionnaire at home visits for 4,861 persons 45–70 years of age, who had lived at least 5 years near any of six major European airports. We assessed noise exposure using detailed models with a resolution of 1 dB (5 dB for United Kingdom road traffic noise), and a spatial resolution of 250 × 250 m for aircraft and 10 × 10 m for road traffic noise.

**Results:**

We found significant exposure–response relationships between night-time aircraft as well as average daily road traffic noise exposure and risk of hypertension after adjustment for major confounders. For night-time aircraft noise, a 10-dB increase in exposure was associated with an odds ratio (OR) of 1.14 [95% confidence interval (CI), 1.01–1.29]. The exposure–response relationships were similar for road traffic noise and stronger for men with an OR of 1.54 (95% CI, 0.99–2.40) in the highest exposure category (> 65 dB; *p*_trend_ = 0.008).

**Conclusions:**

Our results indicate excess risks of hypertension related to long-term noise exposure, primarily for night-time aircraft noise and daily average road traffic noise.

Air traffic continues to increase worldwide, and recent forecasts by the International Air Transport Association (IATA) predict an average annual growth in the number of air passengers of 4.3% until 2015. As a consequence, the airspace is becoming more crowded, particularly in the vicinity of airports, and pollution increases from noise and aircraft exhaust emissions as well as from the associated road traffic.

Hypertension is a major risk factor for coronary heart disease and stroke (Stamler 1992). Recent studies indicate that noise exposure may cause hypertension, but few investigators have studied health effects associated with exposure to aircraft noise (Babisch 2006; van Kempen et al. 2002). Studies carried out around Schiphol (Amsterdam, the Netherlands) Airport in the 1970s showed excess risks of hypertension and other cardiovascular diseases in subjects exposed to high levels of aircraft noise (Knipschild 1977). In a recent study around the same airport, only a slight increase [odds ratio (OR) = 1.2] of self-reported use of cardiovascular drugs was found (Franssen et al. 2004). A Swedish cross-sectional study indicated an exposure–response relation between residential aircraft noise exposure and self-reported (diagnosed by a physician) hypertension (Rosenlund et al. 2001). In a Japanese study near a military air base, there was an exposure–response relationship between aircraft noise and prevalence of hypertension (Matsui et al. 2004).

Noise from road traffic has also been associated with self-reported doctor-diagnosed hypertension (Bluhm et al. 2007) or measured high blood pressure (BP) (Herbold et al. 1989). However, negative results have also been reported (Yoshida et al. 1997). It has been hypothesized that persistent exposure to environmental noise could result in permanent vascular changes, with increased BP and ischemic heart disease as potential outcomes (Stansfeld and Matheson 2003).

The overall evidence suggests that a weak association may exist between long-term noise exposure and hypertension (Babisch 2000; Berglund and Lindvall 1995; Berglund et al. 1999).

The objective of the HYENA (Hypertension and Exposure to Noise near Airports) study was to assess the relationships between exposure to noise generated by aircraft and road traffic near airports and the risk of hypertension.

## Methods

The study complies with the Declaration of Helsinki (World Medical Association 2000) and was approved by ethical committees in all participating centers. Informed written consent was given by all participants before study commencement.

### Participants

The study population included persons 45–70 years of age at the time of interview, with a minimum length of residence of 5 years, living near one of six major European airports [London Heathrow (United Kingdom), Berlin Tegel (Germany), Amsterdam Schiphol (the Netherlands), Stockholm Arlanda (Sweden), Milan Malpensa (Italy), and Athens Elephterios Venizelos (Greece) Airports]. In Stockholm, the population living near City Airport (Bromma) was also included to increase the number of exposed subjects. To maximize exposure contrast, we used a stratified sample of the population based on noise exposure levels. The selection process created exposure contrast to aircraft noise and road traffic noise within countries, ensuring that sufficient numbers of inhabitants in the appropriate age range had expected exposures > 60 dB(A) (A-weighted average sound pressure level) and < 50 dB(A). For the initial selection process of the study population, we used recent aircraft noise contours that were available for all but the new Athens airport, where the information was limited, but we were able to use predicted noise contours calculated in the planning process. We used local noise data to obtain road traffic exposure classification of locations and populations. If such data were unavailable, two simplified methods derived from more complex models were applied. Further details of the selection process can be found elsewhere (Jarup et al. 2005).

### Blood pressure

We used validated and automated BP instruments to minimize observer errors, commonly occurring in the previously used conventional sphygmomanometry (O’Brien et al. 2001). Such instruments are well established in clinical research and are increasing in importance in occupational and environmental medicine (Staessen et al. 2000). Specially trained staff assessed BP three times at home visits; the first measurement was recorded in the beginning of the interview, after 5 min rest, a second BP measurement was recorded after a further 1 min rest in accordance with recommendations of the American Heart Association (Pickering et al. 2005). A third BP reading was taken after the interview (approximately 1 hr) as a validity control. The mean of the first two readings was used to define BP for the subsequent analyses. Using the mean of all three BP readings did not change the results. All BP assessments were performed with the participant in a sitting position. Home visits were distributed over the day as far as feasible, to account for diurnal variations in BP.

Hypertension was defined according to the World Health Organization (WHO 1999, 2003): a systolic BP ≥ 140 or a diastolic BP ≥ 90. In the epidemiologic analyses, we combined the measurements with information on diagnoses of hypertensive disease and medication. The study definition of hypertension included individuals who had either BP levels above the WHO cutoff points or a diagnosis of hypertension (by a physician) in conjunction with use of anti-hypertensive medication, as reported in the interview questionnaire.

### Confounders

We included variables *a priori* considered to be the major potential confounders, being risk factors for hypertension as well as possibly associated with noise exposure. In the adjustment for confounders, country and sex were included as categorized variables; age was included as a continuous variable. We defined alcohol intake as a continuous variable recorded as number of units (1 unit = 10 mL pure ethanol) consumed per week. Body mass index (BMI; weight divided by height squared) was also included as a continuous variable, whereas the level of physical activity was estimated in three categories of exercise by duration only (less than once a week, 1–3 times a week, and > 3 times a week). Education was coded as quartiles of number of years in education, standardized by country means to account for differences in education systems among countries. Smoking is a well known risk factor for heart disease, but not for hypertension, so smoking was not included in the model, as explained further in the “Discussion.”

### Exposure assessment

The Integrated Noise Model (Gulding et al. 1999) served as standard model for aircraft noise and was used in the study areas of Germany, the Netherlands, Sweden, Italy, and Greece to calculate the aircraft noise levels. In the United Kingdom the model Ancon (Ollerhead et al. 1999) was applied; this model fulfills the requirements of the European Civil Aviation Conference (1997).

For road traffic noise, the models used locally were more tailored to the available input data than a centrally prescribed model. We used for the United Kingdom, *Calculation of Road Traffic Noise* (Department of Transport and Welsh Office 1988), for Germany and Italy *Richtlinien für den Lärmschutz an Straßen* [Bundesministerium vor Verkeher (Ministry of Transport) 1990], for Greece and the Netherlands *Standaard Reken- en Meetvoorschrift (SRM)* (Netherlands Ministry of Housing, Spatial Planning and the Environment 2002), and for Sweden the *Nordic Prediction Method* (Bendtsen 1999). We used the *Good Practice Guide for Strategic Noise Mapping* (European Commission 2006) to assess the quality of the input data. The most frequently reported accuracy per input class was 1 dB except for building height, for which less accurate data were obtainable. The spatial resolution (grid size) was 250 × 250 m for aircraft and 10 × 10 m for road traffic noise.

Noise levels for separate periods of the day were modeled for 2002; this year was assumed to be representative for the 5-year period preceding the health status assessment (Jarup et al. 2005). Modeled noise exposure levels were linked to each participant’s home address using geographic information systems technique. For both aircraft and road traffic noise the levels had a 1-dB resolution, except for the United Kingdom, where only 5-dB classes for road traffic noise could be procured. The midpoints of these classes were chosen for the analyses using continuous exposure data.

To assess the effect of noise on hypertension, we used *L*_Aeq,T_ as indicators of exposure as recommended by the WHO (1999). *L*_Aeq,T_ is the A-weighted equivalent continuous noise level over *T* hours. For aircraft noise, the indicators *L*_Aeq,16hr_ (day defined as the hours between 0700 and 2300 or between 0600 and 2200 hours, depending on the local definition) and *L*_night_ (night being defined as the hours between 2300 and 0700 or between 2200 and 0600 hours) were used to differentiate between the effects of daytime and night-time exposure.

In most countries only aggregated 24-hr data on the intensity of road traffic were available. *L*_Aeq,24hr_ and *L*_night_ are derived from these data, and thus highly correlated (overall *r* = 0.97). Consequently, no distinction could be made between the relative effects on hypertension of road traffic noise exposure during the night or during the day.

The accuracy of the noise modeling decreases at lower levels. Input data such as traffic intensities can be so low that relatively small deviations from the actual flows may have large effects on the noise level. To minimize the impact of such inaccuracies on the noise levels, a cutoff value was introduced in several countries at the lower end of the noise levels, based on a local assessment of the accuracy of the input data and noise model characteristics. Because the cutoff value differed among countries, the highest local cutoff value was applied to all data. Noise level values below this cutoff value were assigned the level of the cutoff value. For aircraft noise the cutoff level was for *L*_Aeq,16hr_ 35 dB and for *L*_night_ 30 dB. For road traffic noise the cutoff level was 45 dB for *L*_Aeq,24hr_

### Statistical analysis

Standard statistical methods were applied using standard software packages [e.g. SAS (SAS Institute Inc., Cary, NC, USA), EGRET (Cytel Software Corporation, Cambridge, MA, USA)]. We used logistic regression models with the presence of hypertension as the outcome variable, and exposure variables (categorical and continuous) and confounders as covariates. We calculated 95% confidence intervals (CIs) for each effect estimate. Analyses in 5-dB categories suggested approximately linear relationships, so we used continuous data in the final analyses to increase the statistical power.

To assess the importance of heterogeneity between study sites, we also performed meta-analyses of country-specific analyses, using BioStat Comprehensive Meta-Analysis software (BioStat International Inc., Tampa, FL, USA), using a fixed-effects model.

## Results

A total of 4,861 persons (2,404 men and 2,457 women) between 45 and 70 years of age at the time of interview participated in the study. Participation rates differed among the countries, from approximately 30% in Germany, Italy, and the United Kingdom to 46% in the Netherlands, 56% in Greece, and 78% in Sweden. Participation rates did not differ much among the different noise exposure categories. Overall, response rates were 39, 45, and 45% for aircraft noise categories < 50, 50 to < 65, and ≥ 65 dBA, respectively. The corresponding response rates for road traffic noise were 51, 42, and 37%.

No sex differences were found between responders and nonresponders, and a short nonresponse questionnaire distributed to a sample of nonresponders indicated no obvious differences in prevalence of self-reported hypertension between nonresponders and participants. A minimum of 10% of the questionnaire data were double-entered for all countries. The data entry errors varied among countries, but were generally low (0.13–1.54%).

The sex- and age-adjusted (to the European standard population) prevalence of hypertension was 48.8% in the United Kingdom, 54.6% in Germany, 51.9% in the Netherlands, 52.0% in Sweden, 57.0% in Greece, and 52.1% in Italy.

Table 1 shows the results for the main potential confounders. Country (versus United Kingdom as the baseline; *p* = 0.028), physical activity (duration of exercise; *p* = 0.031), and education (quartiles; *p* = 0.044) were overall statistically significant.

**Figure 1:**
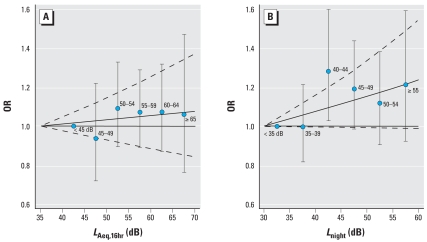
ORs of hypertension in relation to aircraft noise (5-dB categories). *L*_Aeq,16hr_ (*A*) and *L*_night_ (*B*) separately included in the model. Adjusted for country, age, sex, BMI, alcohol intake, education, and exercise. The error bars denote 95% CIs for the categorical (5-dB) analysis. The unbroken and broken curves show the ORs and corresponding 95% CIs for the continuous analysis.

Figure 1 shows the ORs for hypertension in relation to aircraft noise during the day (*L*_Aeq,16hr_) and during the night (*L*_night_). A rise in OR with increasing exposure is indicated primarily for night-time noise. There were no differences in risk between men and women.

**Figure 2:**
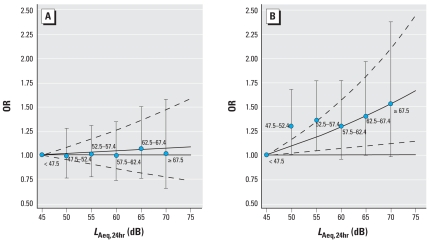
ORs of hypertension in women (*A*) and men (*B*) in relation to road traffic noise (*L*_Aeq,24hr_, 5-dB categories) separately included in the model. Adjusted for country, age, BMI, alcohol intake, education, and exercise. The error bars denote 95% CIs for the categorical (5-dB) analysis. The unbroken and broken curves show the ORs and corresponding 95% CIs for the continuous analysis.

Figure 2 shows the ORs for hypertension in men and women in relation to daily average road traffic noise exposure (*L*_Aeq,24hr_). There was an increase in risk for men related to increasing exposure, but no such trend was found for women. The difference in trend between sexes is statistically significant (*p* = 0.004).

Table 2 shows the ORs for hypertension related to aircraft and road traffic noise using continuous variables after adjustment for the other noise exposure indicators; the ORs show the risk per 10-dB increase in noise exposure. The trends for night-time exposure to aircraft and average 24-hr exposure to road traffic were both statistically significant, whereas 16-hr daytime average aircraft noise exposure was not.

**Figure 3:**
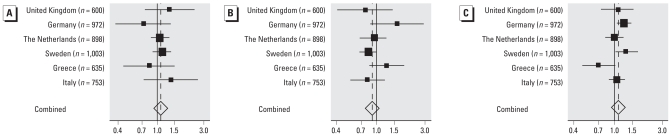
Forest plot showing country-specific ORs for hypertension per 10-dB increase in noise exposure, in relation to (*A*) *L*_night_ and (*B*) *L*_Aeq,16hr_ aircraft noise and (*C*) *L*_Aeq,24hr_ road traffic noise.

We also explored the differences in risks among countries in country-specific analyses, assessing heterogeneity and performing a meta-analysis using a fixed-effects model. As can be seen in Figure 3, there was no obvious heterogeneity among countries for aircraft noise, so pooling the data to gain statistical power is justified. For road traffic noise, there was significant heterogeneity among countries, but the estimated ORs using pooled analyses (adjusted for country) were similar to the computed estimate in the meta-analysis.

## Discussion

The HYENA study is the first to investigate the impact on BP of exposure to noise from aircraft and road traffic near airports. There were significant exposure–response relationships between exposure to night-time aircraft noise exposure, daily average road traffic noise, and risk of hypertension.

There were no significant differences in effect between exposure to noise from aircraft and road traffic (Table 2), although the OR for night-time aircraft noise was somewhat higher than the OR for road traffic noise. It should be noted that all airports but two (Bromma in Sweden and Tegel in Berlin) allow night flights, although some restrictions are in place. However, given the national definitions of *L*_night_ (which are in accordance with the European Environmental Noise Directive) (European Commission 2002), it is clear that there is substantial night-time exposure in all participating countries, particularly, in the “shoulder hours” of the late evening and early morning. The risk of hypertension related to night-time noise exposure tended to be more pronounced than for daytime aircraft noise exposure, although there is slight overlap of CIs, and we cannot exclude some influence on ORs related to collinearity between the two aircraft noise variables (correlation coefficient = 0.8).

The higher risk for night-time noise may be a consequence of less misclassification of exposure during the night (participants are more likely to be at home during the night than during daytime). The higher night-time risks may also be explained by acute physiologic responses induced by night-time noise events that might affect restoration during sleep. Noise-induced instantaneous autonomic responses during sleep do not only occur in waking hours but also in sleeping subjects even when no (electroencephalogram recorded) awakening occurs (Davies et al. 1993). Subjects do not adapt on a long-term basis although a clear subjective habituation occurs after a few nights (Muzet 2002). Repeated arousals from sleep are associated with a sustained increase in daytime BP (Morrell et al. 2000).

Smoking is a well-established risk factor for cardiovascular disease, but its effect on BP is less clear-cut (Green et al. 1986; Narkiewicz et alet al. 2005). BP increases acutely after smoking, so we required that study participants refrained from smoking at least 30 min before BP measurements. To assess whether smoking habits would confound the effects of noise on BP, we initially included smoking in the regression model. However, smoking did not contribute significantly to the model and did not have any impact on the effect estimates of noise, so smoking was not included in the final model.

Risk of hypertension may differ among ethnic groups, although risk patterns are not clear-cut (Sosin et al. 2004). Because ethnicity may also be related to living near airports, we aimed to include ethnicity as a confounding variable. However, ethnic groups differed much among countries, and it was feasible to combine the data only into a crude dichotomous variable (white/nonwhite). The study population was predominantly white (94.4%), and inclusion of the dichotomous ethnicity variable in the analyses did not change the overall risk estimates.

The exposure–response relationship was more pronounced for men exposed to road traffic noise, supporting previous studies that have found excess risk of hypertension for men in relation to road traffic noise (Babisch et al. 2005; Belojevic and Saric-Tanaskovic 2002; Herbold et al. 1989), although the evidence is not fully consistent (Bluhm et al. 2007; von Eiff and Neus 1980). There were no similar sex differences for aircraft noise.

In an attempt to explore whether any sex differences were apparent in retired people (≥ 65 years of age), who may be more likely to spend most of their time at home, we analyzed this subsample of the study population (*n* = 1,076; 546 women and 530 men). We found an excess risk in women for a 10-dB increase in road traffic noise (OR = 1.63; 95% CI, 1.21–2,20), but no significant excess risks for daytime (OR = 1.18; 95% CI, 0.82–1,71) or night-time (OR = 0.91; 95% CI, 0.63–1.34) aircraft noise There were no significant risks in men for any of the noise exposure variables (road traffic noise: OR = 1.03; 95% CI, 0.77–1.38; daytime aircraft noise: OR = 0.96; 95% CI, 0.65–1.43; nighttime aircraft noise: OR = 1.10; 95% CI, 0.73–1.67). The CIs are wide and include the point estimates derived for the total population, apart from women for road traffic noise. This apparent significant excess risk in women may be a result of less misclassification of exposure but could, of course, also be a chance finding. Further research is needed to clarify the reason for the sex differences in risk related to (road traffic) noise exposure.

A potential weakness of our study is the low response rate in most of the participating countries. However, a descriptive analysis indicated only minor differences between participants and nonresponders in distribution between aircraft noise exposure categories. However, for road traffic noise, contrary to what might have been expected, response rates were lower in the high exposure category; any potential bias related to this is difficult to assess, but is unlikely to be substantial. The response rates for road traffic noise categories in particular are rather crude, because they are based on estimates from the selection procedure (Jarup et al. 2005). Furthermore, there were no apparent differences in the prevalence of hypertension between participants and nonresponders. It is unlikely that health outcomes such as hypertension would give rise to a selection bias, potentially resulting in falsely increased risks (Franssen et al. 2004).

Our results show differences in the prevalence of hypertension among participating countries, the United Kingdom having the lowest prevalence (48.8%) and Greece the highest (57.0%). Our prevalence rates are in general higher than previously published data, although differences are difficult to interpret because of differences in population age structure (Kearney et al. 2005). However, relations between country prevalence are similar to the data published by Kearney et al. (2005), apart from Greece, which has a markedly lower prevalence in the previously published paper.

In conclusion, the HYENA study found statistically significant effects on BP of nighttime aircraft noise and average 24-hr road traffic noise exposure, the latter for men in particular. Hypertension is an important independent risk factor for myocardial infarction and stroke, and the increased risk of hypertension in relation to aircraft and road traffic noise near airports demonstrated in our study may therefore contribute to the burden of cardiovascular disease. Our results indicate that preventive measures should be considered to reduce road traffic noise and nighttime noise from aircraft.

## Members of the HYENA Study Team

Maria Chiara Antoniotti

Regional Agency for Environmental Protection, Piedmont Region, Grugliasco, Italy

Ageliki Athanasopoulou

National and Kapodistrian University of Athens, Athens, Greece

Giorgio Barbaglia

Regional Agency for Environmental Protection, Piedmont Region, Grugliasco, Italy

Alessandro Borgini

Regional Agency for Environmental Protection, Piedmont Region, Grugliasco, Italy

Elli Davou

National and Kapodistrian University of Athens, Athens, Greece

Matteo Giampaolo

Regional Agency for Environmental Protection, Piedmont Region, Grugliasco, Italy

Jessica Kwekkeboom

The National Institute of Public Health and Environmental Protection (RIVM), Bilthoven, the Netherlands

Birgitta Ohlander

Institute of Environmental Medicine, Karolinska Institutet, Stockholm, Sweden

Salvatore Pisani

Regional Agency for Environmental Protection, Piedmont Region, Grugliasco, Italy

Joy Read

Imperial College London, London, United Kingdom

Yousouf Soogun

Imperial College London, London, United Kingdom

Yvonne Tan

Imperial College London, London, United Kingdom

Eva Thunberg

Institute of Environmental Medicine, Karolinska Institutet, Stockholm, Sweden

Gabriele Wölke

Federal Environmental Agency, Berlin, Germany

Venetia Velonakis

National and Kapodistrian University of Athens, Athens, Greece

Yannis Zahos

National and Kapodistrian University of Athens, Athens, Greece

## Figures and Tables

**Table 1:** ORs (95% CIs) of hypertension in relation to the main confounders.

Variable	OR (95% CI)	*p-*Value
Germany (vs. UK)	1.34 (1.07–1.69)	0.012
The Netherlands (vs. UK)	1.30 (1.03–1.63)	0.027
Sweden (vs. UK)	1.44 (1.15–1.80)	0.002
Greece (vs. UK)	1.42 (1.10–1.83)	0.007
Italy (vs. UK)	1.22 (0.95–1.56)	0.118
Age	1.07 (1.06–1.08)	< 0.001
Sex (female vs. male)	0.67 (0.59–0.76)	< 0.001
Alcohol intake	1.01 (1.00–1.02)	0.001
BMI	1.11 (1.10–1.13)	< 0.001
Exercise, 1–3 times a week vs. < once a week	0.96 (0.81–1.15)	0.681
Exercise, > 3 times a week vs. < once a week	0.82 (0.71–0.95)	0.009
Education quartile 2 vs. 1	1.01 (0.83–1.23)	0.897
Education quartile 3 vs. 1	0.81 (0.68–0.98)	0.027
Education quartile 4 vs. 1	0.83 (0.69–1.00)	0.049

Country, age, sex, BMI, and alcohol intake, physical activity, and exercise simultaneously included in the model.

**Table 2:** ORs (95% CIs) of hypertension related to aircraft and road traffic noise using continuous variables, showing the risk per 10 dB increase in noise exposure.

Variable	OR (95% CI)	*p*-Value
*L*_Aeq,16hr_ aircraft	0.928 (0.829–1.038)	0.190
*L*_night_ aircraft	1.141 (1.012–1.286)	0.031
*L*_Aeq,24hr_ road traffic	1.097 (1.003–1.201)	0.044

All noise indicators were included in the model, which was adjusted for country, age, sex, BMI, alcohol intake, education, and exercise.
