# Correction: Hypertension and Exposure to Noise Near Airports: the HYENA Study

**DOI:** 10.1289/ehp.10775-erratum

**Published:** 2008-06-01

In the statistical analysis section of “Hypertension and Exposure to Noise Near Airports: the HYENA Study” [Environ Health Perspect 116:329–333 (2008)], Jarup et al. erroneously named Biostat International (Tampa, FL) as the manufacturer of the meta-analysis software used in the study. The software is actually produced by Biostat (Englewood, NJ).

The authors regret the error.

